# Effects of DHA-Rich n-3 Fatty Acid Supplementation and/or Resistance Training on Body Composition and Cardiometabolic Biomarkers in Overweight and Obese Post-Menopausal Women

**DOI:** 10.3390/nu13072465

**Published:** 2021-07-19

**Authors:** Elisa Félix-Soriano, Alejandro Martínez-Gayo, María José Cobo, Adriana Pérez-Chávez, Javier Ibáñez-Santos, Natalia Palacios Samper, Iñaki Goikoetxea Galarza, Marta Cuervo, Marisol García-Unciti, Pedro González-Muniesa, Silvia Lorente-Cebrián, María J. Moreno-Aliaga

**Affiliations:** 1 Department of Nutrition, Food Science and Physiology, School of Pharmacy and Nutrition, University of Navarra, 31008 Pamplona, Spain; efelix@alumni.unav.es (E.F.-S.); amartinez.104@alumni.unav.es (A.M.-G.); mcobo@unav.es (M.J.C.); aperez.39@alumni.unav.es (A.P.-C.); mcuervo@unav.es (M.C.); mgarcia@unav.es (M.G.-U.); pgonmun@unav.es (P.G.-M.); slorente@alumni.unav.es (S.L.-C.); 2 Center for Nutrition Research, School of Pharmacy and Nutrition, University of Navarra, 31008 Pamplona, Spain; 3 Studies, Research and Sports Medicine Centre (CEIMD), Government of Navarre, 31005 Pamplona, Spain; javier.ibanez.santos@navarra.es (J.I.-S.); nataliapalacioss@gmail.com (N.P.S.); goiko92@gmail.com (I.G.G.); 4 IdISNA, Navarra Institute for Health Research, 31008 Pamplona, Spain; 5 CIBER Physiopathology of Obesity and Nutrition (CIBERobn), Carlos III Health Institute (ISCIII), 28029 Madrid, Spain

**Keywords:** postmenopause, obesity, DHA, resistance training, glucose tolerance, body composition, lipid metabolism

## Abstract

Resistance training (RT) and n-3 polyunsaturated fatty acids (n-3 PUFA) supplementation have emerged as strategies to improve muscle function in older adults. Overweight/obese postmenopausal women (55–70 years) were randomly allocated to one of four experimental groups, receiving placebo (olive oil) or docosahexaenoic acid (DHA)-rich n-3 PUFA supplementation alone or in combination with a supervised RT-program for 16 weeks. At baseline and at end of the trial, body composition, anthropometrical measures, blood pressure and serum glucose and lipid biomarkers were analyzed. Oral glucose tolerance tests (OGTT) and strength tests were also performed. All groups exhibit a similar moderate reduction in body weight and fat mass, but the RT-groups maintained bone mineral content, increased upper limbs lean mass, decreased lower limbs fat mass, and increased muscle strength and quality compared to untrained-groups. The RT-program also improved glucose tolerance (lowering the OGTT incremental area under the curve). The DHA-rich supplementation lowered diastolic blood pressure and circulating triglycerides and increased muscle quality in lower limbs. In conclusion, 16-week RT-program improved segmented body composition, bone mineral content, and glucose tolerance, while the DHA-rich supplement had beneficial effects on cardiovascular health markers in overweight/obese postmenopausal women. No synergistic effects were observed for DHA supplementation and RT-program combination.

## 1. Introduction

Menopause is a critical stage in the physiological process of aging among women, with final menstrual period being a marker of aging and health [1], and age at menopause influencing the risk for all-cause mortality [2]. During menopause, redistribution of fat mass from gluteo-femoral depots towards the visceral cavity, alongside with muscle and bone mass loss, give rise to a constellation of unfavorable metabolic conditions such as insulin resistance, unhealthy lipid profiles, abnormal glucose metabolism and decreased metabolic rate [3]. Altogether, these circumstances mimic those of aging in a short period of time and increase the risk of developing sarcopenic obesity, metabolic syndrome, Type 2 diabetes mellitus, coronary heart disease and osteoporosis, which are more prevalent diseases among post- than pre-menopausal women, and in older women than men [3,4,5].

On the other hand, obesity can also have a negative impact in the menopausal transition, and thus in the process of aging, as obesity itself increases the risk for such metabolic diseases and also for frailty [6]. Although interrelationships between obesity, menopause and aging are not established yet, several interventions have been developed with the aim to improve health and well-being among the older, obese population. Interestingly, some authors have described sedentary lifestyles as the main factor to affect health and well-being in older subjects [7]. Hence, exercise training interventions have been developed, with resistance training (RT) as a novel approach to increase muscle strength and lean mass, with the consequent improvement in physical function and metabolic profile, together with preventing future frailty and disability in older adults [8,9].

RT can elicit a potent neuromuscular stimulus that, when maintained on a regular basis, is able to improve lean mass, muscle strength, bone mineral density, and physical function also among postmenopausal women [8,10,11,12]. Such improvements in muscle metabolism are the main cause for RT ability to improve glucose homeostasis in older women [13]. However, effects on insulin resistance are yet to be elucidated, and RT has been established to be effective in insulin resistant, but not in healthy, older subjects [13,14]. Likewise, hypotensive effects have been limited to normotensive older subjects in some studies [15], while heterogenous effects have been highlighted for both hypertensive and normotensive older women depending on their response to RT [16]. Concerning lipid metabolism, some studies have revealed an effect on lowering total cholesterol (total chol) and LDL-cholesterol (LDL-chol) while increasing HDL-cholesterol (HDL-chol) [17,18], and others have shown neutral effects [15] or pointed out the high variability in individuals responses to RT [19]. Body composition results are also inconclusive, and it seems that only long periods of RT can elicit changes in fat mass and muscle mass [20], and so well designed interventions studying RT programs have found no effects on body composition, even when strength improvements were found [11,12,21]

Among dietary interventions, n-3 polyunsaturated fatty acids (n-3 PUFA) eicosapentaenoic acid (EPA) and docosahexaenoic acid (DHA) have been recently discovered to play a role in muscle protein synthesis [22]. This finding has prompted the development of interventions with both RT and n-3 PUFA in postmenopausal women [23,24,25,26]. Nevertheless, such studies have failed to investigate n-3 PUFA combined with RT effects further than strength gains or muscle protein synthesis. Noteworthy, DHA has been suggested to have more beneficial effects on obesity than EPA [27,28]. Interestingly, higher DHA levels in plasma lipids but not EPA or alpha-linolenic acid are related with lower progression of coronary artery disease in postmenopausal women [29]. Moreover, DHA levels are higher in pre than post-menopausal women [30] and have been demonstrated to have higher antithrombotic effects than EPA [31]. DHA supplementation has been proved to lower triglycerides (TG) in a dose-dependent manner in healthy postmenopausal women [32]. Although DHA effects on lowering LDL-chol have not been demonstrated in this population, effects on lowering small, dense LDL-chol percentage have been reported [33], and also on increasing HDL-chol [31]. Moreover, meta-analyses have highlighted the role of DHA and EPA on lowering blood pressure in the general population [34]. Remarkably, DHA levels are lower also in postmenopausal Type 2 diabetes mellitus patients than in their healthy counterparts [30], and it has been highlighted that n-3 PUFA might be effective in improving insulin sensitivity in individuals under metabolic risk [35]. However, DHA effects in post-menopausal women are still controversial regarding insulin sensitivity, as well-designed trials have shown no effects [36]. Regarding body composition they seem to have no relevant effects [37] except for bone mineral density, which has been found to be positively associated to the n-3 PUFA content in erythrocytes in osteoporotic, postmenopausal women [38].

Thus, the objective of this study was to examine if supplementation with a DHA-rich fish oil concentrate and a progressive RT program, alone or in combination, for 16 weeks, could have beneficial effects on improving body composition, lipid, and glucose metabolism biomarkers, as well as muscle strength and quality in overweight and obese postmenopausal women.

## 2. Materials and Methods

### 2.1. Participants

A total of 124 postmenopausal women were recruited by advertisement in local newspapers and by phone calls to volunteers from the database of the Metabolic Unit (MU) of the University of Navarra. The inclusion criteria were being 55–70 years old and overweight II/obese Type I (BMI of 27.5–35 kg/m^2^), with a stable weight in the last 3 months (±3 kg) and an overall physical and physiological condition in accordance with the aim of the study (i.e., not suffering from musculoskeletal injuries that limited the subject performance during the RT program). Exclusion criteria for enrolment were as follows: use of some regular prescription medication, including hormonal therapy, oral antidiabetic drugs, hypolipidemic drugs, and proton pump inhibitors. Antihypertensive therapy, thyroid hormones, anxiolytic, and antidepressant therapies were also included as exclusion criteria if dosage had been modified in the three months prior to the screening visit and/or the start of the trial; as well as to suffer from any severe metabolic, hepatic, renal, cardiovascular, neuromuscular, arthritic, pulmonary or other debilitating diseases; or to follow any special diets in the three months prior to the start of the trial. Volunteers were also excluded if they had suffered from eating disorders, surgically treated obesity, or if they had a history of alcohol or drug abuse.

Before inclusion in the study, all candidates were thoroughly screened using an extensive medical history (including blood biochemical data), resting electrocardiogram, and blood pressure measurements, at the MU of the University of Navarra. Participants were informed in detail about the possible risks and benefits of the study and gave their written informed consent prior to being enrolled in the study. The intervention was approved by the Research Ethics Committee of the University of Navarra (140/2015mod2) and was performed in compliance with the Helsinki Declaration guidelines [39]. The study was registered at clinicaltrials.gov as NCT03300388.

### 2.2. Study Design

The study was designed as a randomized double-blind placebo-controlled trial (RCT), in which participants were allocated into four parallel intervention groups for 16 weeks: (1) the placebo group (P) received placebo capsules containing olive oil (6 capsules of 0.5 g), (2) the omega-3 group (n-3) received DHA-rich fish oil concentrate capsules providing 1650 mg/day of DHA and 150 mg/day of EPA as ethyl esters, with a total content of 1950 mg/day of n-3 PUFA, distributed in 6 capsules of 0.5 g of fish oil concentrate each, (3) the placebo + resistance training group (P+RT) received 6 placebo capsules and followed a progressive RT program of 2 sessions/week, and (4) the omega-3 + resistance training group (n-3+RT) received the 6 DHA-rich fish oil capsules containing 1650 mg/day of DHA and 150 mg/day of EPA, and followed a progressive RT program of 2 sessions/week.

### 2.3. Nutritional Intervention

Once the screening was completed, volunteers were randomly allocated to one of the four groups using the software platform MATLAB^®^ (The Mathworks™, Natick, MA, USA). Randomization criteria were age and BMI according to World Health Organization classification. Thus, the volunteers were randomized to create similar groups depending on whether they belonged to a group of age classified as adult or older adult (55–59 and 60–70 years old, respectively) [40]; and a BMI of overweight Grade II or obesity Type I (27.5–29.9 and 30–35 kg/m^2^, respectively) [41].

At baseline and at the end of the trial, participants attended the MU at the University of Navarra in 8–12 h fasting conditions, where anthropometric measurements, body composition data and blood pressure determinations were carried out by a dietitian and a nurse. Basal fasting blood samples were then extracted in order to obtain serum/plasma to measure biochemical parameters, and an oral glucose tolerance test (OGTT) was carried out as described previously [42]: 75 g of anhydrous glucose (GlycoSull^®^, Química Clínica Aplicada, Tarragona, Spain) were given to the volunteer and blood samples were extracted at 30′, 60′, 90′ and 120′.

At the end of the baseline visit, volunteers were given written dietary recommendations based on the guidelines from the Spanish Society for Communitarian Nutrition (SENC, 2016) [43]. Follow up dietary consultations were scheduled every two weeks, and dietary patterns were evaluated with a validated questionnaire of 14 items to assess adherence to the Mediterranean diet (p14) [44] and a food frequency questionnaire (FFQ) [45] at baseline and at the end of the study, in order to evaluate potential changes along the intervention.

When baseline visit was completed, volunteers were also given the corresponding supplements. Subjects were asked to report any secondary effect to evaluate its possible association with capsules consumption. Thus, once the baseline visit of the trial was completed and in every follow-up visit, all intervention groups received two boxes containing 6 blisters with 10 capsules each, for a total of 120 capsules. Participants were asked to return boxes in every follow-up visit to evaluate adherence to supplementation by leftover pill count.

Physical activity (PA) was also controlled with a validated PA questionnaire [46] filled by participants at baseline and endpoint study visits. To compare PA between the four study groups also with a direct measure, participants were asked to wear an accelerometer (ActiGraph GT3X, Actigraph Corporation, Pensacola, FL, USA), during a random and complete week of the study. The accelerometer was programmed for the subject’s gender, age, weight, height, race and worn position in the body. The participants were instructed to not change their habitual physical activity habits during the 16 weeks of the trial.

### 2.4. Supplements Information

Participants consumed two capsules with each meal (breakfast, lunch, and dinner). Both placebo and DHA-rich fish oil concentrate (DHA 55%) capsules (DHA^scc^ premium) were provided by Solutex^®^ (Madrid, Spain). The DHA capsules contained tocopherol extracts as antioxidants to protect the highly unsaturated fatty acids from oxidation and small amounts of silicon dioxide as stabilizer. The same quantity of tocopherols was added to the olive oil capsules, although the monounsaturated fatty acids in olive oil are expected to be more resistant to oxidation. The low amount of the other stabilizer included was not expected to have any significant effect or modify the actions of fish oil concentrate on health benefits. To guarantee that the DHA-rich fish oil-derived supplements were not oxidized, peroxide and anisidine values were tested during the study and were below maximum. Olive and fish oils were provided in hard gelatin transparent liquid fill capsules and were similar in shape and size. Only a small difference in the thickness/color of the oils could be appreciated.

The dose of DHA-rich fish oil-derived supplement was selected based in previous studies [47,48,49], and in accordance to the U.S. Food and Drug Administration (FDA) recommendations of not exceeding 3 g/day EPA and DHA, with up to 2 g/day from dietary supplements [50]. To fulfill these criteria, the consumption of fish was controlled depending on their n-3 PUFA’s composition according to the European Food Safety Authority (EFSA) recommendations for normal cardiac function (250 mg/day), based on food composition tables from Mataix-Verdú et al. [51] and online food composition databases (Easy Diet^®^ and Odimet^®^ software, Spain). Consumption of n-3-PUFA enriched food and dietary supplements was not allowed during the study. Although the EFSA considers safe long-term consumption of EPA and DHA supplements at combined doses of up to about 5 g/day [52], more restrictive FDA criteria were applied and therefore, not exceeding 3 g/daily intake of EPA and DHA were allowed/considered for this trial. Fatty acid composition of the olive oil used as placebo was analyzed as described by Ansorena et al. [53] and it is shown in Table S1.

### 2.5. Resistance Training Program

After the baseline visit was completed, subjects allocated in the RT groups were asked to assist to the Studies, Research and Sports Medicine Centre training facilities (CEIMD), twice a week during 16 weeks of intervention, to perform dynamic resistance exercise [54,55]. Eight exercises for upper and lower main muscular groups were included in the training program. Two routines were designed with six exercises each: leg press, chest press, knee extension and lat pulldown were maintained along the RT program, while shoulder press and hip extension (Routine 1) and chest fly and leg curl (Routine 2) were selected to complete each routine, changing every two weeks. Before testing and training, subjects attended three sessions for familiarization with the procedure of voluntary force production.

Strength tests were performed at the beginning, midst, and at the end of the trial to obtain strength gains/losses data and to adjust training loads to each volunteer’s strength. In this study, the 1-repetition maximum (1-RM) approach was used for testing [56]. Training progression was established using the pyramidal training approach, so as 50% of intensity was selected to start the training program, and a maximum intensity of 80% was reached at week 10 [57]. Three to four series were performed in each training session with 8–15 repetitions adapting to training loads. In each session, one of the researchers was present to direct and assist each subject towards ensuring adequate performance in each exercise (work rates, loads and ranges of motion) following American College of Sports Medicine (ACSM) guidelines for older adults.

To control for strength gains/losses also in untrained groups, first and last follow-up visits were scheduled at the training facilities for subjects allocated to these groups in order to perform 1-RM tests with its corresponding familiarization session. Relative strength was calculated by dividing the maximum weight lifted in the 1-RM test (kg) to the subjects’ body weight (kg) for leg press and chest press exercises. Muscle quality was expressed according to Pina et al. [58] as the ratio of the maximum weight lifted in the 1-RM tests (kg) to lean soft tissue (kg) of the lower and upper limbs.

### 2.6. Evaluation of Weight Loss and Body Composition

The main outcome of the study was the reduction of fat mass. Body composition was analyzed at baseline and at the end of trial by total and segmented dual X-ray absorptiometry (Lunar iDXA, encore 14.5, Madison, WI, USA), as previously reported [59]. Legs and arms lean soft tissue mass changes were used as estimators of their muscle mass changes, as previously described in aging [60] and after exercise [61]. In addition, anthropometric measurements were obtained including arm, waist, hip, thigh, and calf circumference, as well as arm, thigh, and calf skinfolds, following the ISAK guidelines at baseline and end of the trial [62].

### 2.7. Evaluation of Lipid and Glucose Metabolism and other Biomarkers

Once basal blood samples were extracted, they were centrifuged at 1500× *g* for 15 min at 4 °C and aliquots of serum/plasma were frozen at −80 °C until analysis. Fasting serum lipid and glucose metabolism biomarkers, including total chol, HDL-chol, TG, glucose, and OGTT timepoints’ glucose levels were determined on an autoanalyzer (Pentra C-200; HORIBA ABX, Madrid, Spain) following manufacturer’s instructions at baseline and at the end of the trial. LDL-chol was calculated using the Friedewald equation. Fasting insulin was determined with an ELISA kit (#10-1132-01, Mercodia, Uppsala, Sweden) following the manufacturer’s instructions on an autoanalyzer (Triturus ELISA Instrument, Grifols, Barcelona, Spain). Indexes for insulin resistance HOMA-IR and Triglycerides to Glucose (TyG) index for insulin resistance were calculated as described previously [42].

### 2.8. Statistical Analysis

Considering fat mass losses as the primary outcome, based on the results reported by previous studies on the placebo untrained group and the n-3 trained group [63,64], the estimated effect size was 1.185. Taking a bilateral alpha of 95% and a power calculation of 90%, the number of volunteers per group was 16. Considering a 25% of drop-out rate, the estimated number of subjects per group would be 20.

Statistical analyses were performed using STATA, version 14. Data were expressed as mean ± SD, and differences were significant at two-sided *p* value < 0.05. Possible confounding variables were used for adjustment, and values were expressed as mean (SEM). To select the appropriate test, normal distribution was assessed using Shapiro–Wilk test and Breush–Pagan/Cook–Weisberg heteroscedasticity test. Comparisons between groups at baseline were evaluated by a one-factor ANOVA test or Kruskal–Wallis test. The comparison between baseline and endpoint within each group were assessed by paired Student’s *t*-test or Wilcoxon signed-rank test as appropriate.

Due to the factorial design of the study, the statistical test two-way ANOVA was selected to analyze if the changes observed after the intervention were significantly different due to one of the two study factors, DHA-rich n-3 PUFA supplementation, RT, or by an interaction between both. The results of the test were represented with the *p* value (*p* < 0.05) for significant effects, or as ns for the non-significant ones, appearing below the corresponding factor. Factors were named as n-3 for the DHA-rich n-3 PUFA supplementation, RT for the exercise program, and n-3xRT for the interaction.

When statistical significance appeared at the interaction level (n-3xRT) contrasts were performed with the aim to differentiate the group effects. If not, the significant main effects were studied, which must be considered as (i) a main effect for supplementation (n-3) differentiating the changes observed in the placebo groups from those observed in DHA-supplemented groups, whether they were allocated to exercise or not; and (ii) a main effect for exercise (RT) differentiating the changes observed in the exercised vs. the non-exercised groups, whether they were supplemented with DHA or not.

## 3. Results

### 3.1. Basal Characteristics and Flowchart of the Participants

**Figure 1:**
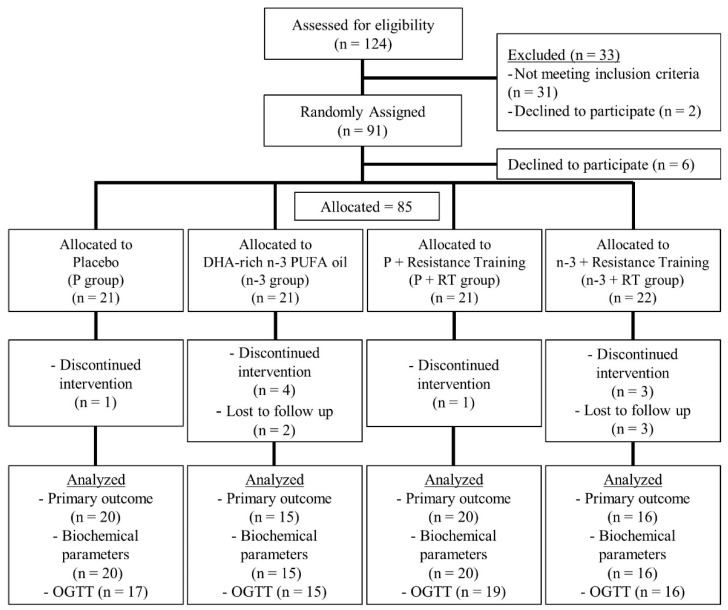
Flowchart of participants from the screening to the endpoint visit of the study (16 weeks). In total, 85 out of the 91 women who met the inclusion criteria started the intervention. Further, 14 participants did not complete the study (16.5% drop out), as they either discontinued follow-up due to unexpected health problems (*n* = 5, 3 unrelated to the study and 2 related to capsules consumption), time incompatibilities (*n* = 2), withdrew from the study (*n* = 3), or were not compliant with the training sessions (*n* = 4). There were two dropouts in the n-3 group probably related to capsules consumption, one of them was related to gastroesophageal reflux and the other one related to itch in the hands. Dropout rates were 4.8% for P and P+RT groups, 28.6% for n-3 group and 27.3% for n-3+RT group. For Oral Glucose Tolerance Test (OGTT) analyses, 3 subjects were excluded due to problems with venous insertion of the catheter in the P group, and 1 subject was excluded due to lack of measure at one timepoint of the glucose excursion curve in the P+RT group. DHA: Docosahexaenoic Acid; PUFA: Polyunsaturated Fatty Acids.

Of the 124 volunteers screened for the intervention, 85 initiated the trial and 71 finished the study (Figure 1). Baseline characteristics of the study subjects were similar between the four experimental groups, except for the basal glycemia, that was moderately lower in the P group than in the P+RT and n-3+RT groups (Table S2). All the groups also exhibited a similar adherence to the Mediterranean diet pattern and similar dietary fat intake and n-6/n-3 PUFA ratio. 

Although all groups reported a reduction in total fat intake at the end of the trial, no significant changes were observed between the four intervention groups (Table S3). Moreover, all groups showed similar PA levels, as estimated by validated questionnaires and measured by accelerometry. Furthermore, the intervention groups did not significantly change their PA pattern during the trial, rather than the RT expected in the allocated groups (Table S3). Finally, the mean adherence to the RT program and supplementation (capsules intake) was above 95% at the end of the intervention in all groups (Figure S1).

### 3.2. Effects on Whole Body Composition and Anthropometric Measurements

After the 16 weeks of intervention, all groups showed a moderate but statistically significant reductions in body weight, BMI, and the percentage of fat mass (Table 1). Visceral fat mass was also significantly reduced after the intervention in all groups except for n-3+RT group, but a reduction was observed when adjusted for total weight loss. Interestingly, lean mass percentage increased in the four groups after the intervention. However, the analysis of the changes between groups by two-way ANOVA revealed no significant statistical differences between the four experimental groups for any of the previously described body composition parameters.

Noteworthy, bone mineral content (BMC) significantly decreased in those groups that were not allocated to the RT program (Table 1). In fact, the analysis of the changes between groups revealed that the RT program was able to significantly prevent this reduction in BMC observed in the untrained groups (Table 1).

Regarding anthropometric measurements, all groups showed a decrease in waist, and hip circumferences (Table 1), but the waist/hip ratio was significantly reduced only in the P and n-3 groups. Statistical differences were maintained in P group and appeared in the n-3+RT group when adjusting for weight loss, while they disappeared in the n-3 group (Table 1). When looking for differences in changes between groups due to RT and/or n-3-PUFA supplementation, no significant differences were found (Table 1).

### 3.3. Effects on Segmented Body Composition and Anthropometric Measurements

Fat and lean mass composition as well as anthropometric measurements of arms and legs were studied. A significant reduction in arms weight was observed only in untrained groups after the trial. Arms lean mass tended to decrease in the non-trained groups. 

Thus, the two-way ANOVA analysis suggested that the RT program significantly prevented the lean (muscle) mass and arms weight loss compared to the untrained groups (Table 2). Arms fat mass was reduced in the four intervention groups, although it did not reach statistical significance in the n-3+RT group. The analysis of changes between groups showed that the reduction in arms fat mass was significantly lower in the groups receiving n-3 PUFA supplementation (Table 2). Nevertheless, arms circumference and tricipital skinfold were reduced in all groups after the intervention, without significant differences for RT or n-3-PUFA supplementation when changes between groups were analyzed (Table S4).

Regarding lower body composition, legs weight was reduced in all intervention groups (Table 2). Although reductions tended to be higher in RT vs. untrained groups, two-way ANOVA did not reach statistical significance (*p* = 0.098). Legs lean mass did not change in any of the intervention groups, neither when baseline-endpoint nor when changes between groups were analyzed. Nevertheless, legs fat mass did decrease in the four groups of study, with a significantly higher decrease in the RT vs. the non-trained groups. Anthropometric measurements reflected similar results, as calf circumference was reduced in all study groups, with significantly higher losses only in RT groups when changes between groups were compared (Table 2). Thigh circumference was also moderately reduced in the four experimental groups but did not change significantly by either RT or n-3-PUFA supplementation when analyzed by two-way ANOVA. Similar to calf circumference, calf and thigh skinfolds reductions were significantly higher in the RT groups compared to the untrained groups (Table 2).

### 3.4. Effects on Muscle Strength and Quality

**Figure 2:**
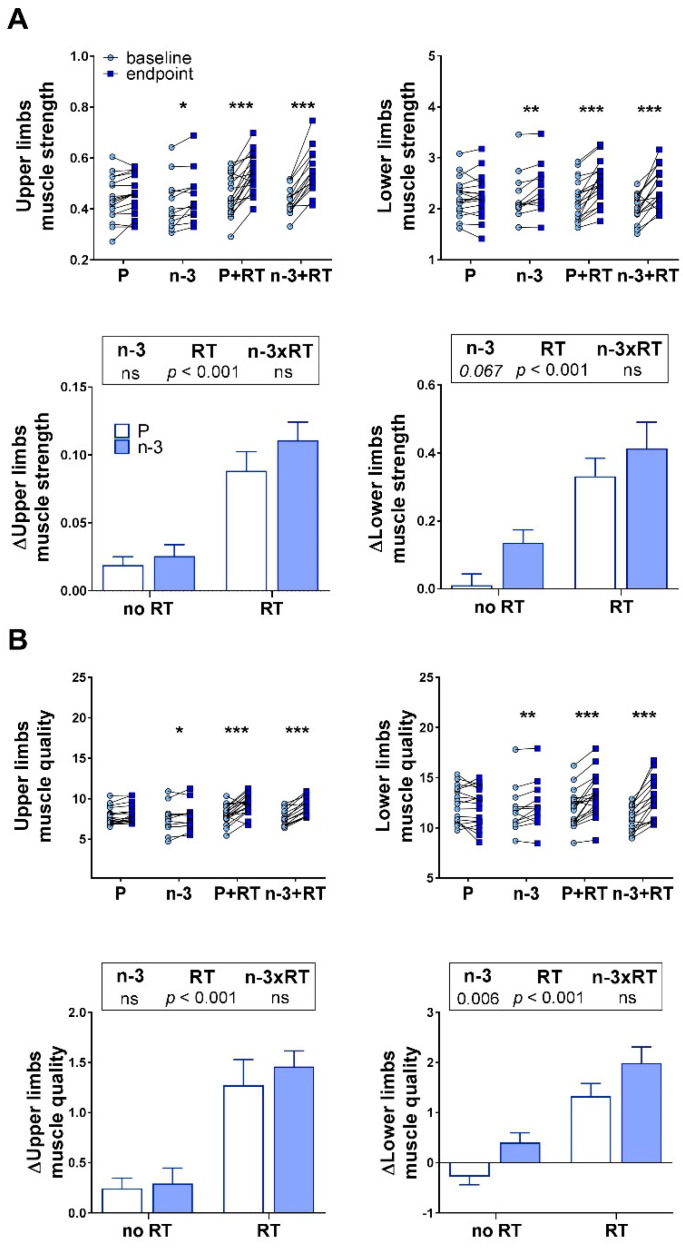
Effects of 16 weeks of DHA-rich n-3 PUFA (n-3) supplementation and/or resistance training (RT) on upper limbs (left panels) and lower limbs (right panels) muscle strength (**A**) and muscle quality (**B**). Muscle strength was calculated as 1-RM (kg)/body weight (kg); and muscle quality, as 1-RM (kg)/local lean mass (kg). Baseline-endpoint differences (joined dots graphs) were studied by paired Student’s *t*-test or Wilcoxon’s signed-rank test after testing for normality. *** *p* < 0.001; ** *p* < 0.01; * *p* < 0.05 vs. baseline. Differences in changes between groups (bar graphs) were compared by two-way ANOVA. When a significant effect was found for one of the main factors of study, the *p* value was represented under the corresponding factor in the legend appearing above the graph. Factors were named as n-3 for DHA-rich n-3 PUFA supplementation, RT for exercise, and n-3xRT for the interaction between both. Statistical significance for a factor establishes a main effect for such factor on differentiating the groups it classifies (n-3: n-3-supplemented vs. P-supplemented groups; RT: RT vs. no-RT groups) according to the studied parameter (ns, nonsignificant, *p* > 0.05). Data are mean ± SEM. P: placebo group, n-3: DHA-rich n-3 PUFA supplemented group; P+RT: placebo + resistance training group; n-3+RT: DHA-rich n-3 PUFA supplemented + resistance training group.

Muscle strength values (kg, 1-RM tests) were normalized to the subjects’ body weight at baseline and at the end of the trial. Likewise, muscle quality was calculated as previously described [58] for both upper and lower limbs, by dividing muscle strength (kg lifted in the chest and leg press 1-RM tests)/lean mass (kg measured in arms and legs DXA segmented analyses). As expected, the RT groups significantly increased their muscle strength and quality (*p* < 0.001) compared to the untrained groups, both in upper and lower limbs (Figure 2). 

Noteworthy, the DHA-rich supplement did not influence muscle strength but revealed a tendency to promote this effect in lower limbs (*p* = 0.067) that could rely on the local improvement in muscle quality (*p* < 0.01). However, these effects observed in lower limbs by n-3 PUFA supplementation were not mimicked by the results observed in the upper limbs. No synergistic effects were observed for the DHA-rich supplementation on strength and muscle quality gains derived from the RT program.

### 3.5. Effects on Blood Pressure and Lipid Metabolism Biomarkers

With respect to blood pressure measurements, systolic and diastolic blood pressure did not show significant changes in any group except for a significant reduction in diastolic blood pressure in the n-3+RT group (Table 3). 

However, two-way ANOVA revealed a significant effect for the DHA-rich supplement on lowering diastolic blood pressure in n-3 compared to P-supplemented groups. TG were significantly reduced after the intervention in all groups, except the P group. 

The analysis of the differences of changes between groups showed that this reduction in TG was significantly higher in the n-3 supplemented groups as compared with those receiving placebo (Table 3). Total chol and LDL-chol were reduced only in P+RT group after the intervention, but when values were adjusted for fat mass loss and the corresponding baseline value, this effect was also observed in the P group. However, when comparing the differences between groups, no statistical differences were found for the changes in cholesterol parameters (Table 3).

### 3.6. Effects on Serum Glucose Metabolism Biomarkers

Fasting glucose tended to decrease in all intervention groups, but without statistical significance in any of them when comparing baseline-endpoint values (only a significant reduction was observed in the n-3+RT group after adjusting by changes in fat mass and values at baseline). However, no statistically differences were observed when changes between groups were compared by two-way ANOVA (Table 3). 

Fasting insulin and HOMA-IR index were significantly reduced in P-supplemented and n-3+RT groups after the intervention (after adjusting by changes in fat mass and values at baseline, a significant decrease in fasting insulin and HOMA-IR was also observed in the P+RT group). However, no significant differences induced by RT or n-3-PUFA supplementation were observed when the changes between groups were analyzed (Table 3). The TyG index, a reliable marker for insulin resistance, was significantly reduced after the intervention in both groups performing the RT program (P+RT and n-3+RT); yet, when the differences in changes between the intervention groups were evaluated, no significant effects were reached (Table 3).

**Figure 3:**
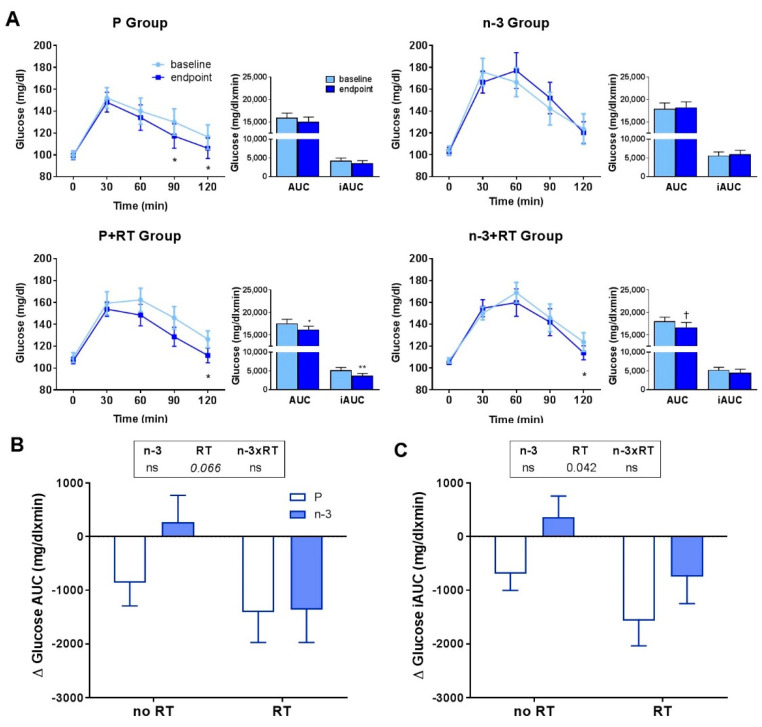
Effects of 16 weeks of DHA-rich n-3 PUFA (n-3) supplementation and/or resistance training (RT) on (**A**) oral glucose tolerance (OGTT) excursion curves, AUC and iAUC at baseline and endpoint, and (**B**,**C**) AUC and iAUC changes. Comparisons between baseline and endpoint values (**A**) were assessed using paired Student’s t-test or Wilcoxon’s signed-rank test. * *p* < 0.05, ** *p* < 0.01, † *p* = 0.060 vs. baseline. Comparison of changes in AUC (**B**) and iAUC (**C**) between groups was evaluated by two-way ANOVA. When a significant effect was found for one of the main factors of study, the *p* value was represented under the corresponding factor in the legend appearing above the graph. Factors were named as n-3 for DHA-rich n-3 PUFA supplementation, RT for exercise, and n-3xRT for the interaction between both. Statistical significance for a factor establishes a main effect for such factor on differentiating the groups it classifies (n-3: n-3-supplemented vs. P-supplemented groups; RT: RT vs. no-RT groups) according to the studied parameter (ns, nonsignificant, *p* > 0.05). Data are mean ± SEM. P: placebo group, n-3: DHA-rich n-3 PUFA supplemented group; P+RT: placebo + resistance training group; n-3+RT: DHA-rich n-3 PUFA supplemented + resistance training group; AUC: area under curve, iAUC: incremental AUC.

The effect of a 16-week RT program alone or in combination with DHA supplementation on glucose tolerance was also studied through an OGTT. For that, a high-glucose beverage (75 g) was administered to the volunteers in order to evaluate the changes in serum glucose. The glucose excursions after OGTT are represented in Figure 3A, which shows the levels before and after the intervention in the four study groups. After the intervention, all groups, except the n-3 group, exhibited a better response to the OGTT than at baseline. However, when the areas under the curves (AUC) were calculated, only a significant reduction when comparing before and after was found in the P+RT group, and a tendency to decrease was observed in n-3+RT group. To adjust for basal glucose, iAUC was also calculated, with significant reductions in the P+RT group. When differential changes between groups were analyzed by two-way ANOVA, the AUC showed a tendency to decrease in RT groups (*p* = 0.066), and a significant decrease in iAUC was found in RT groups vs. not trained groups (Figure 3B,C). Nevertheless, the DHA supplementation had no remarkable effect on any of the glucose tolerance parameters analyzed.

## 4. Discussion

Here, we describe the effects of a 16-week intervention with a DHA-rich supplement combined or not with a RT program on body composition, serum glucose and lipid metabolism biomarkers, blood pressure and muscle strength and quality in overweight and obese postmenopausal women. While few previous studies in postmenopausal women have combined n-3 PUFA supplementation and RT, these trials were focused on muscle metabolism, strength and function; and some lacked either a placebo group, or an untrained group [23,24,25,26].

In the present study, the four experimental groups showed moderate but significant reductions in body weight, BMI, and fat percentage, along with lower visceral adipose tissue, and waist and hip circumferences after the intervention. These results suggest the efficacy of dietary advice for a healthy diet on remodeling body composition without a hypocaloric dietary approach. It should be considered that several studies have suggested that olive oil supplementation, the placebo used in our trial, can also promote moderate weight loss [65]. However, the amount of olive oil supplemented (3.0 g/day) was equivalent to approximately less than 10% daily recommendations for the Spanish population (45 g/day) [43]. Therefore, it is unlikely that the olive oil-based placebo supplement might have contributed to the unexpected weight loss observed in the placebo groups.

On the other hand, our data in the RT groups may apparently contrast with previous studies observing effects for RT on inducing fat mass loss in adults as compared to non-trained subjects [15,19]. However, RT effects in postmenopausal women without dietary treatment only had such effects in body composition when longer intervention periods [20,66], higher training volumes [9,23,26], groups mixing women and men [14,19,67,68], or only overweight or few proportions of obese women were included [18,66,69]. Even in those circumstances, no effects in body composition have been reported in postmenopausal women [11,12,13].

The lack of effects for RT on whole-body muscle mass, namely fat free mass or lean mass, has been reported in postmenopausal women [11,12,13,21]. Similar to those showing effects on adiposity, those reporting effects on increasing muscle mass used rather long exercise protocols, applied higher training volumes, mixed men and women, or were performed only in overweight subjects [9,19,25,70,71]. Here, we provide evidence of a 16-week protocol that achieved muscle strength and quality gains, alongside high adherence rates (above 95%), with a moderate RT program (10 out of 16 weeks with 70–80% RM loads) in previously untrained, overweight, and obese post-menopausal women. Others have reported increased muscle strength, quality, cross sectional area or muscle protein synthesis that were not translated to higher muscle mass in older populations nor in postmenopausal women [9,12,13,72]. Noteworthy, Churchward-Venne et al. [73] compared two different training programs in older overweight women and concluded that, although there are no non-responders to exercise, the time-effect response is highly individualized. Similarly, Ahtiainen et al. [19] collected studies of their group developing the same RT program in different populations and observed a large interindividual variations in the training response that could not be explained by sex, age, body composition or nutritional status. Therefore, the physiological adaptations secondary to RT might rely on individual features whose causative role are beyond the purpose of this study.

Analyses of upper and lower body composition by DXA segmented analyses revealed principal effects for the RT program when studying changes among groups. In upper limbs, our RT program suggested effects on arms muscle hypertrophy and weight maintenance compared to the untrained groups, who showed a moderate weight and lean mass losses. To our knowledge, it is the first time these effects of RT compared to untrained postmenopausal women are reported in the literature with shorter training programs, as improvements in segmented muscle mass have been reported previously only for overweight older women and longer training periods and/or higher frequencies [20,58]. Regarding lower body composition, RT did not show an effect in legs weight and lean (muscle) mass, but it did increase fat mass loss as compared to untrained groups. Such effects could be explained by women’s natural distribution of adiposity in gluteofemoral areas, and thus could be the main effect of our RT program on fat mass losses. In fact, those studies reporting increases in lower limbs lean mass after RT were conducted in leaner women [21], and no differences have been reported on lower limbs fat mass [21,23,58]. Anthropometric measurements results agreed with those found in DXA for lower limbs body composition, as RT had a significant effect in decreasing leg skinfolds and calf circumference as compared to untrained groups. On the contrary, thigh circumference did not change with RT, indicating a possible hypertrophy in thighs and higher fat mass losses at calves. Upper limbs anthropometric measurements did match those of DXA regarding arms hypertrophy with RT, as arms circumference did not change. Thus, these results support that segmented body composition changes measured by DXA were comparable to those obtained with the anthropometric measurements including the observed upper limbs hypertrophy and lower limbs fat mass loss after RT. Moreover, these results establish anthropometry as a reliable tool for evaluating changes in upper and lower body composition in overweight/obese postmenopausal women under exercise programs.

Findings of RT effects improving upper limbs muscle (lean) mass, as well as upper and lower limbs muscle quality and strength were consistent with resistance training improving also whole-body BMC. A clear effect of RT on BMC maintenance was observed, despite small changes and high variability. However, there were no effects on bone mineral density (BMD) (data not shown). Data of exercise programs effects on bone metabolism in postmenopausal women are large, and thus several meta-analyses have been conducted, concluding that combined RT and high impact exercises are the best to maintain BMD after longer interventional periods [10], while local effects of RT on BMD have also been observed [74]. Although BMC results can be found in trials, such results were not included in the statistical analyses of such meta-analyses. In fact, BMC has less clinical relevance compared to BMD, and thus studies of BMC changes in postmenopausal women after RT are lacking. Despite this, both BMD and BMC are predictors of fracture risk [75]. BMC improvements without concomitant effects on BMD after RT in postmenopausal women with low bone mass have been reported before [76]. Thus, our finding supports a beneficial effect of RT on bone mass independent of n-3-PUFA supplementation and regardless of having or not low BMD and BMC in a population at risk of bone mass loss. In fact, recent studies investigating the effects of RT in postmenopausal women revealed local increments in femoral BMC that occurred together with an increase in bone thickness [77]. In turn, bone thickness is strongly related to an increment in the femoral neck fracture load [78], which is defined as the load which is great enough to break the bone, and thus could be understood as bone resistance to fracture. Furthermore, BMC has even been proposed for the clinical diagnose of osteoporosis besides BMD [79], being the first a proxy of bone geometry and the latter of bone quality, both relevant for the maintenance of a healthy bone structure.

As expected, no effects were observed for n-3 PUFA supplementation alone or combined with RT on body composition changes [37]. Other groups have observed similar results to ours using DHA and EPA at similar doses (1.62 g/day, 1.9:1 DHA:EPA) [48,49]. Such studies did not show differences between the placebo (6 capsules x 1g Sunola oil per day) and fish oil supplemented group in any of the body weight and composition parameters after 12 weeks of supplementation [48], neither when combining it with a very low energy diet [49]. Surprisingly, a significant effect of n-3-PUFA supplementation was found for smaller fat mass losses in arms than placebo supplementation. Conversely, these effects were not translated to smaller subcutaneous fat mass losses, as both tricipital skinfold and arm circumference decreased in the four groups of study without differences in changes. In fact, it has been described that the main synergistic effects of n-3 PUFA to RT on body composition are the promotion of muscular protein synthesis together with beneficial effects on the neuromuscular system [24,80], with no further effects on body composition. With this regard, our results showed effects for RT on increasing muscular strength and quality significantly in lower and upper limbs, while n-3 PUFA revealed an effect on muscle quality in lower limbs. Such effects are supported by those observed by Rodacki et al. [26] and Strandberg et al. [25], who showed increased activation and neuromuscular response to RT in groups supplemented with n-3-PUFA (2 g, EPA 29.5 ± 0.7% and DHA 23.6 ± 0.2%, for 90 or 150 days) or with a n-3 PUFA enriched diet (ratio n-6/n-3 < 2, 24 weeks) respectively, coupled to a RT program. It must be noted that their RT programs used high training intensities for longer training periods (80% of 1-RM for 10 and 20 weeks), and that subjects were leaner than those included in our study, according to BMI (mean BMI ~24.7–27.7 kg/m^2^).

The DHA-rich n-3 PUFA supplementation exerted two remarkable effects in main cardiovascular risk factors, blood pressure and circulating lipids. Firstly, diastolic blood pressure was reduced by the DHA-rich supplementation when compared to the placebo supplementation. This result is in line with others who have observed inverse associations between erythrocyte DHA content and hypertension in postmenopausal women [33,81]. Although a recent trial in middle aged women showed no results after n-3 PUFA supplementation with similar DHA dose to those used in our study, a smaller sample size (n = 6, 1600 mg DHA + 400 mg EPA) was studied [82]. On the other hand and similar to what has been demonstrated in older subjects and in postmenopausal women [33,83], the DHA-rich n-3 PUFA supplementation also induced a reduction in circulating TG, which is in agreement with the recognized n-3 PUFA hypotriglyceridemic claimed effects. Concerning total chol, LDL-chol and HDL-chol no effects were observed for the DHA-rich supplementation on improving their levels when compared to the placebo groups, nor when comparing within groups. Accordingly, a recent meta-analysis concluded that n-3 PUFA exert effects only in lowering TG [83]. These effects of DHA supplementation on cardiovascular risk factors are especially relevant in the postmenopausal population, in which cardiovascular-mortality exhibits a sudden rise [84].

Regarding the RT program, no effects were observed on any of the cardiovascular risk parameters when comparing trained groups to untrained groups, although circulating levels of Total chol, LDL-chol and HDL-chol were decreased in the P+RT group, and both trained groups revealed a decrease in TG. Despite the recent meta-analyses describing beneficial effects of RT on circulating lipid levels in adults [15,85], multiple trials investigating RT effects on blood lipid levels observed no effects in postmenopausal women and older adults [19,21,68,86]. Thus, several studies have assessed this question by examining the individual response to RT in older adults. Although the occurrence of non-responders to RT was discarded recently [73,87], the individual response to RT was quantified in the aforementioned studies and described to be highly heterogeneous, leading to a great variability in the measured outcomes especially in blood lipids [19,73], as observed in our n-3+RT group. On the other hand, the presence of subjects with delayed time-response effect might have led to little changes in body composition [67,73], that in turn mediate circulating lipid levels [69,88]. Nevertheless, it seems that the most consistent outcome of RT in blood lipid levels in older adults and postmenopausal women is the increase in HDL-chol, even when the rest of lipids do not change [19,67,89]. By contrast, our HDL-chol levels were decreased in both trained groups and significantly in P+RT group, possibly due to the higher levels at baseline observed in our participants compared to those participating in the aforementioned trials. Moreover, such levels could be a consequence of increased levels of the atherogenic HDL-chol fraction.

A remarkable effect exerted by RT was the improvement in glucose tolerance. Thus, RT groups showed an effect in OGTT-iAUC compared to untrained groups, regardless of n-3-PUFA supplementation. These effects of RT on improving glucose tolerance in postmenopausal women can rely on the local increases in muscle (lean) mass and the muscle strength and quality gains. In fact, sarcopenic older subjects under RT exhibited lower glucose AUC after the intervention [90], demonstrating muscle’s essential role in glucose metabolism. However, the improved glucose tolerance was not accompanied by similar outcomes in fasting glucose, insulin, and HOMA-IR index, although small but significant improvements were observed in TyG index for insulin resistance in both trained groups. Insulin sensitivity measurements are lacking in this study, but it might be the causative factor for the improved glucose metabolism and TyG index, regardless of fasting insulin and HOMA-IR levels, and secondary to the increased glucose tolerance. In fact, RT has been shown to have lower effects on insulin resistance as compared to aerobic or combined (resistance + aerobic) training [14] which are, in turn, demonstrated to elicit higher fat mass losses [91]. Moreover, the higher variability in responses to the intervention observed in the n3-PUFA+RT group may have blunted both RT and n-3 PUFA principal effects when changes between groups were studied. Nevertheless, it has to be noted that lack of synergistic effects for RT and fish oil supplementation on fasting glucose and insulin was also observed in postmenopausal women in the study of Da Boit et al. [24].

There are limitations in the current study that need to be considered. Some previous clinical trials with n-3 PUFA derived from fish oil have reported difficulties to successful blinding because of the fishy taste and odor of these fatty acids [92]. We must report a difficulty with the double blinding of our study, due to the fishy taste reported by some of the participants allocated in the DHA-rich supplement groups and a small difference in the thickness/color of the olive oil (placebo) and the fish oil concentrate, as they were provided in transparent liquid fill capsules. Although some of the investigators providing the capsules to the volunteers suspect about the type of supplements, most of the participants were blinded concerning the type of supplement they were receiving. Moreover, the researchers in charge to carry out the biochemical analysis of the blood samples and DXA analysis were totally blinded and therefore we consider that this minor incident with the blinding has not affected the results reported in this study.

Another limitation of the study is that we had few participants between 65–70 years, and the mean ages of subjects were closer to the middle-age, and thus interrelationships between obesity and menopause may have had a more relevant role than expected in the response to both RT and n-3-PUFA supplementation. Moreover, evidence in the current literature for both RT and n-3-PUFA interventions in postmenopausal women is largely heterogenous regarding methodologies conducted and characteristics of the study populations, making it difficult to compare effects between studies. These facts and the applicability of the findings obtained in the present study highlight the relevance of performing future trials involving a higher number of postmenopausal older women.

## 5. Conclusions

In summary, our data suggest that progressive intensity RT has beneficial effects on upper limbs muscular hypertrophy and lower limbs fat mass loss, on muscle strength and muscle quality, along with whole body BMC maintenance and improved glucose tolerance in postmenopausal women. The DHA-rich oil supplement had the previously documented effect on lowering fasting TG levels and lowered diastolic blood pressure. However, no effects were found on insulin resistance or other biomarkers of lipid metabolism, and no relevant synergistic effects for n-3-PUFA and RT were observed.

## Figures and Tables

**Table 1:** Effects of 16 weeks of DHA-rich n-3 PUFA (n-3) supplementation and/or resistance training (RT) on whole body composition and anthropometric measures in overweight/obese post-menopausal women.

	P	n-3	P+RT	n-3+RT	Two-Way ANOVA ^c^		
N	20	15	20	16	n-3	RT	n-3xRT
Age (years)	58.75 ± 3.39	58.00 ± 2.78	58.95 ± 3.46	58.13 ± 3.14			
Weight (kg)							
Baseline	76.75 ± 4.99	80.34 ± 8.51	77.76 ± 7.92	80.57 ± 6.60			
Change	−2.66 ± 2.95 ^a,^***	−2.65 ± 2.47 ^a,^**	−2.21 ± 2.39 ^a,^***	−2.70 ± 3.49 ^a,^**	ns	ns	ns
BMI (kg/m^2^)							
Baseline	30.25 ± 2.30	30.39 ± 1.94	30.79 ± 2.34	31.07 ± 1.82			
Change	−1.07 ± 1.16 ^b,^**	−1.03 ± 0.94 ^a,^***	−0.90 ± 0.94 ^a,^***	−1.06 ± 1.34 ^a,^**	ns	ns	ns
Fat mass (%)							
Baseline	47.44 ± 3.42	45.55 ± 2.38	47.05 ± 3.96	46.70 ± 2.90			
Change	−2.27 ± 1.14 ^a,^***	−1.58 ± 1.34 ^a,^***	−1.77 ± 1.50 ^a,^***	−2.12 ± 2.40 ^a,^**	ns	ns	ns
Visceral fat (kg)							
Baseline	1.30 ± 0.44	1.37 ± 0.44	1.27 ± 0.51	1.18 ± 0.47			
Change	−0.20 ± 0.19 ^a,^***	−0.11 ± 0.14 ^a,^**	−0.11 ± 0.18 ^b,^*	−0.12 ± 0.23 ^a,^^†^	ns	ns	ns
Adjusted change ^d^	−0.20(0.04) ^a,^***	−0.11(0.02) ^a,^***	−0.12(0.03) ^b,^**	−0.12(0.03) ^a,^**	ns	ns	ns
Lean mass (%)							
Baseline	49.76± 3.20	51.52 ± 2.18	50.16 ± 3.78	50.53 ± 2.76			
Change	2.21 ± 1.08 ^a,^***	1.49 ± 1.26 ^a,^***	1.70 ± 1.43 ^a,^***	2.01 ± 2.34 ^a,^**	ns	ns	ns
BMC (g)							
Baseline	2152.65 ± 308.05	2366.33 ± 332.76	2156.10 ± 231.21	2240.31 ± 258.36			
Change	−27.60 ± 17.36 ***	−17.53 ± 20.22 ^b,^**	1.40 ± 30.33	−1.38 ± 32.81	ns	0.001	ns
Adjusted change ^d^	−25.95(4.19) ***	−17.50(5.08) ^b,^**	−2.08(6.63)	−1.26(7.10)	ns	*p* < 0.001	ns
Waist circumference (cm)							
Baseline	93.11 ± 4.57	95.00 ± 7.63	92.67 ± 5.47	93.90 ± 7.16			
Change	−3.45 ± 2.62 ^a,^***	−3.15 ± 2.94 ^a,^**	−3.01 ± 1.80 ^a,^***	−4.04 ± 3.73 ^a,^***	ns	ns	ns
Adjusted change ^d^	−3.35(0.26) ^a,^***	−3.18(0.65) ^a,^***	−3.08(0.38) ^b,^***	−4.03(0.62) ^a,^***	ns	ns	ns
Hip circumference (cm)							
Baseline	110.68 ± 7.14	112.50 ± 5.78	110.65 ± 5.74	113.35 ± 6.82			
Change	−2.40 ± 2.97 ^a,^**	−3.00 ± 3.12 ^a,^**	−3.17 ± 4.99 ^a,^*	−3.06 ± 3.52 ^a,^**	ns	ns	ns
Adjusted change ^d^	−2.42(0.37) ^a,^***	−2.87(0.57) ^a,^***	−3.33(1.17) ^a,^*	−2.89(0.91) ^a,^**	ns	ns	ns
Waist/hip ratio							
Baseline	0.84 ± 0.04	0.85 ± 0.06	0.84 ± 0.06	0.83 ± 0.08			
Change	−0.01 ± 0.01 ^a,^***	−0.01 ± 0.01 ^a,^*	−0.00 ± 0.04	−0.02 ± 0.03	ns	ns	ns
Adjusted change ^d^	−0.01(0.00) ^a,^**	−0.01(0.00)	−0.00(0.01)	−0.02(0.01) ^a,^*	ns	ns	ns

DHA: Docosahexaenoic Acid; PUFA: Polyunsaturated Fatty Acids; P: placebo group; n-3: DHA-rich n-3 PUFA supplemented group; P+RT: placebo + resistance training group; n-3+RT: DHA-rich n-3 PUFA supplemented + resistance training group; BMI: body mass index; BMC: bone mineral content. Data are mean ± SD. ^a^ Paired Student’s *t*-test, ^b^ Wilcoxon’s signed-rank test. ^c^ Differences between groups for changes were evaluated by two-way ANOVA. The *p* value for the main factors of study, supplementation (named as n-3), exercise (named as RT), and the interaction between both (named as n-3xRT) appears under the corresponding column. Statistical significance for a factor establishes a main effect for such factor on differentiating the groups it classifies (n-3: n-3-supplemented vs. P-supplemented groups; RT: RT vs. no-RT groups), according to the studied parameter. ^d^ Means (SEM) adjusted by changes in body weight. *** *p* < 0.001, ** *p* < 0.01, * *p* < 0.05, ^†^ *p* trend (*p* = 0.056–0.061) vs. baseline; ns, nonsignificant (*p* > 0.05).

**Table 2:** Effects of 16 weeks of DHA-rich n-3 PUFA (n-3) supplementation and/or resistance training (RT) on segmented body composition and anthropometric measures in overweight/obese postmenopausal women.

	P	n-3	P+RT	n-3+RT	Two-Way ANOVA ^c^		
N	20	15	20	16	n-3	RT	n-3xRT
Arms weight (kg)							
Baseline	8.65 ± 0.89	8.60 ± 1.17	8.73 ± 1.21	8.83 ± 1.04			
Change	−0.37 ± 0.40 ^a,^***	−0.33 ± 0.42 ^a,^**	−0.13 ± 0.40	0.01 ± 0.60	ns	0.010	ns
Adjusted change ^d^	−0.38(0.07) ^a,^***	−0.32(0.11) ^a,^*	−0.15(0.09)	0.02(0.11)	ns	0.003	ns
Arms fat mass (kg)							
Baseline	4.36 ± 0.60	4.01 ± 0.68	4.32 ± 0.68	4.24 ± 0.71			
Change	−0.33 ± 0.27 ^a,^***	−0.24 ± 0.24 ^b,^**	−0.22 ± 0.28 ^a,^**	−0.11 ± 0.41	ns	ns	ns
Adjusted change ^d^	−0.34(0.04) ^a,^***	−0.23(0.06) ^a,^**	−0.24(0.05) ^a,^***	−0.10(0.07)	0.041	ns	ns
Arms lean mass (kg)							
Baseline	4.02 ± 0.56	4.30 ± 0.60	4.13 ± 0.66	4.29 ± 0.47			
Change	−0.04 ± 0.18	−0.09 ± 0.22	0.09 ± 0.19 ^a,†^	0.13 ± 0.30	ns	0.002	ns
Adjusted change ^d^	−0.05(0.04)	−0.09(0.06)	0.09(0.04) ^a,†^	0.13(0.07) ^a,†^	ns	0.002	ns
Legs weight (kg)							
Baseline	25.10 ± 3.59	27.52 ± 3.28	26.55 ± 3.91	27.11 ± 3.95			
Change	−0.60 ± 1.79 ^b,†^	−0.91 ± 0.97 ^a,^**	−0.95 ± 1.12 ^a,^**	−0.98 ± 1.45 ^a,^*	ns	ns	ns
Adjusted change ^d^	−0.51(0.22) ^b,^**	−0.86(0.10) ^a,^***	−1.08(0.22) ^a,^***	−0.91(0.10) ^a,^***	ns	ns	ns
Legs fat mass (kg)							
Baseline	11.29 ± 2.58	12.16 ± 2.38	12.36 ± 2.88	12.18 ± 2.47			
Change	−0.62 ± 0.90 ^b,^**	−0.73 ± 0.66 ^a,^***	−0.96 ± 0.73 ^a,^***	−0.97 ± 1.02 ^a,^**	ns	ns	ns
Adjusted change ^d^	−0.60(0.12) ^b,^**	−0.70(0.08) ^a,^***	−1.02(0.14) ^a,^***	−0.92(0.12) ^a,^***	ns	0.005	ns
Legs lean mass (kg)							
Baseline	13.03 ± 1.57	14.51 ± 1.66	13.40 ± 1.64	14.11 ± 1.88			
Change	0.02 ± 0.98	−0.16 ± 0.50	0.02 ± 0.57	0.02 ± 0.63	ns	ns	ns
Adjusted change ^d^	0.01(0.14)	−0.15(0.10)	−0.05(0.13)	0.03(0.99)	ns	ns	ns
Thigh circumference (cm)							
Baseline	56.42 ± 4.54	56.75 ± 3.48	59.71 ± 5.21	60.42 ± 6.42			
Change	−1.49 ± 1.75 ^b,^**	−0.84 ± 1.18 ^a,^*	−2.38 ± 3.00 ^a,^**	−1.50 ± 2.52 ^a,^*	ns	ns	ns
Adjusted change ^d^	−1.07(0.24) ^b,^**	−0.91(0.40) ^a,^*	−2.71(0.57) ^a,^***	−1.60(0.56) ^a,^*	ns	ns	ns
Calf circumference (cm)							
Baseline	38.05 ± 2.12	39.59 ± 2.58	40.15 ± 2.41	39.30 ± 2.55			
Change	−0.32 ± 0.54 ^a,^*	−0.39 ± 0.67 ^a,^*	−0.81 ± 0.78 ^b,^***	−0.68 ± 0.69 ^a,^**	ns	0.017	ns
Adjusted change ^d^	−0.26(0.09) ^a,^*	−0.38(0.15) ^b,^*	−0.85(0.19) ^a,^***	−0.68(0.13) ^a^^,^***	ns	0.005	ns
Thigh skinfold (mm)							
Baseline	39.92 ± 4.76	41.25 ± 5.49	42.67 ± 2.99	41.06 ± 5.37			
Change	0.43 ± 3.36	−1.23 ± 2.28 ^b,^*	−3.43 ± 3.15 ^a,^***	−5.19 ± 7.61 ^b,^**	ns	*p* *< 0.001*	ns
Adjusted change ^d^	−0.28(0.61)	−0.70(0.54) ^b,^*	−3.31(0.66) ^a^^,^***	−4.64(1.92) ^b,^*	ns	*p* *< 0.001*	ns
Calf skinfold (mm)							
Baseline	31.07 ± 5.28	32.02 ± 5.60	33.26 ± 4.88	34.76 ± 5.45			
Change	−1.26 ± 3.65	−2.51 ± 3.05 ^a,^**	−5.17 ± 4.63 ^a,^***	−7.73 ± 7.40 ^b,^**	ns	*p* *< 0.001*	ns
Adjusted change ^d^	−1.46(0.70) ^a,†^	−2.25(0.80) ^a,^*	−5.05(1.92) ^a,^***	−7.44(1.82) ^b,^**	ns	*p* *< 0.001*	ns

DHA: Docosahexaenoic Acid; PUFA: Polyunsaturated Fatty Acids; P: placebo group; n-3: DHA-rich n-3 PUFA supplemented group; P+RT: placebo + resistance training group; n-3+RT: DHA-rich n-3 PUFA supplemented + resistance training group. Data are mean ± SD. ^a^ Paired Student’s *t*-test, ^b^ Wilcoxon’s signed-rank test. ^c^ Differences between groups for changes were evaluated by two-way ANOVA. The *p* value for the main factors of study, supplementation (named as n-3), exercise (named as RT), and the interaction between both (named as n-3xRT) appears under the corresponding column. Statistical significance for a factor establishes a main effect for such factor on differentiating the groups it classifies (n-3: n-3-supplemented vs. P-supplemented groups; RT: RT vs. no-RT groups), according to the studied parameter. ^d^ Means (SEM) adjusted by changes in body weight. *** *p* < 0.001, ** *p* < 0.01, * *p* < 0.05, ^†^ *p* trend (*p* = 0.052–0.061) vs. baseline; ns, nonsignificant (*p* > 0.05).

**Table 3:** Effects of 16 weeks of DHA-rich n-3 PUFA (n-3) supplementation and/or resistance training (RT) on blood pressure and glucose and lipid metabolism biomarkers in overweight/obese postmenopausal women.

	P	n-3	P+RT	n-3+RT	Two-Way ANOVA ^c^		
N	20	15	20	16	n-3	RT	n-3xRT
SBP (mm Hg)							
Baseline	121.83 ± 19.68	119.18 ± 7.98	122.73 ± 14.96	123.67 ± 9.01	ns	ns	ns
Change	−2.40 ± 10.84	0.20 ± 10.96	−1.22 ± 14.45	−6.00 ± 11.79	ns	ns	ns
DBP (mm Hg)							
Baseline	80.04 ± 12.38	79.71 ± 6.05	79.08 ± 7.88	81.62 ± 6.67	ns	ns	ns
Change	−1.40 ± 7.13	−2.09 ± 6.97	1.83 ± 7.05	−4.94 ± 7.70 ^a,^*	0.035	ns	ns
TG (mg/dL)							
Baseline	92.64 ± 29.47	118.20 ± 55.31	110.90 ± 51.66	101.34 ± 33.26			
Change	1.94 ± 26.29	−28.87 ± 52.97 ^b,^*	−17.10 ± 23.78 ^b,^*	−18.88 ± 28.73 ^b,^*	0.047	ns	ns
Adjusted change ^d^	2.99(6.14)	−22.09(7.34) ^a,^**	−13.98(4.02) ^a^^,^**	−20.75(5.06) ^a,^**	0.038	ns	ns
Total Chol (mg/dL)							
Baseline	237.40 ± 30.79	239.73 ± 46.41	254.50 ± 27.83	250.31 ± 45.89			
Change	−8.10 ± 25.20	−9.00 ± 44.86	−21.45 ± 24.34 ^b,^**	−14.41 ± 43.32	ns	ns	ns
Adjusted change ^d^	−12.66(4.87) ^a^^,^*	−12.66(8.06)	−17.42(3.83) ^a,^***	−10.41(8.62)	ns	ns	ns
LDL−Chol (mg/dL)							
Baseline	153.31 ± 32.65	154.20 ± 36.89	168.40 ± 24.49	164.90 ± 44.04			
Change	−6.98 ± 19.29	−4.22 ± 35.87	−14.20 ± 23.09 ^b,^**	−8.14 ± 38.54	ns	ns	ns
Adjusted change ^d^	−10.46(3.65) ^a^^,^*	−7.79(7.47)	−10.69(3.95) ^a,^*	−5.29(7.60)	ns	ns	ns
HDL−Chol (mg/dL)							
Baseline	65.74 ± 16.77	61.89 ± 16.38	63.92 ± 14.61	65.15 ± 11.03			
Change	−0.55 ± 9.45	−0.99 ± 13.97	−3.83 ± 8.32 ^a,^^†^	−2.50 ± 9.46	ns	ns	ns
Adjusted change ^d^	−0.08(1.70)	−0.07(2.68)	−4.35(1.83) ^a,^*	−1.63(2.08)	ns	ns	ns
Glucose (mg/dL)							
Baseline	98.57 ± 13.03	103.90 ± 15.72	109.14 ± 18.90	108.35 ± 11.32			
Change	−1.83 ± 9.22	−0.86 ± 11.87	−3.59 ± 20.78	−4.73 ± 9.36 ^a,†^	ns	ns	ns
Adjusted change ^d^	−3.60(2.82)	−2.53(2.67)	−1.33(1.52)	−3.83(1.52) ^a,^*	ns	ns	ns
Insulin (mU/L)							
Baseline	10.02 ± 4.41	9.90 ± 5.21	9.49 ± 5.00	10.54 ± 4.05			
Change	−2.63 ± 3.91 ^b,^*	−0.84 ± 2.75	−1.35 ± 4.03	−1.96 ± 2.76 ^b,^*	ns	ns	ns
Adjusted change ^d^	−2.28(0.63) ^a^^,^**	−1.01(0.75)	−1.83(0.63) ^a^^,^**	−1.75(0.49) ^a,^*	ns	ns	ns
HOMA−IR index							
Baseline	2.51 ± 1.43	2.48 ± 1.19	2.67 ± 1.86	2.82 ± 1.14			
Change	−0.71 ± 1.05 ^b,^*	−0.23 ± 0.87	−0.54 ± 1.52	−0.60 ± 0.74 ^b,^**	ns	ns	ns
Adjusted change ^d^	−0.63(0.15) ^a^^,^***	−0.40(0.21) ^a,†^	−0.56(0.17) ^a,^**	−0.53(0.13) ^a,^**	ns	ns	ns
TyG index							
Baseline	8.45 ± 0.45	8.64 ± 0.41	8.64 ± 0.47	8.59 ± 0.39			
Change	−0.05 ± 0.31	−0.15 ± 0.37	−0.17 ± 0.27 ^a,^*	−0.23 ± 0.33 ^a,^*	ns	ns	ns
Adjusted change ^d^	−0.08(0.07)	−0.13(0.09)	−0.16(0.06) ^a^^,^*	−0.22(0.06) ^a,^**	ns	ns	ns

DHA: Docosahexaenoic Acid; PUFA: Polyunsaturated Fatty Acids; P: placebo group, n-3: DHA-rich n-3 PUFA supplemented group; P+RT: placebo + resistance training group; n-3+RT: DHA-rich n-3 PUFA supplemented + resistance training group; SBP: systolic blood pressure; DBP: diastolic blood pressure; TG: triglycerides; Total Chol: total cholesterol; HDL-Chol: HDL-cholesterol; LDL-Chol: LDL-cholesterol; TyG: triglycerides to glucose index. Data are mean ± SD. ^a^ Paired Student’s *t*-test, ^b^ Wilcoxon’s signed-rank test. ^c^ Differences between groups for changes were evaluated by two-way ANOVA. The *p* value for the main factors of study, supplementation (named as n-3), exercise (named as RT), and the interaction between both (named as n-3xRT) appears under the corresponding column. Statistical significance for a factor establishes a main effect for such factor on differentiating the groups it classifies (n-3: n-3-supplemented vs. P-supplemented groups; RT: RT vs. no-RT groups), according to the studied parameter. ^d^ Means (SEM) adjusted by changes in fat mass and values at baseline. *** *p* < 0.001, ** *p* < 0.01, * *p* < 0.05, ^†^ *p* trend (*p* = 0.053–0.062) vs. baseline; ns, nonsignificant (*p* > 0.05).

## Supplementary Materials

The following are available online at https://www.mdpi.com/article/10.3390/nu13072465/s1, Figure S1: adherence to supplementation and exercise in the four intervention groups, Table S1: fatty acid composition of the olive oil capsules, Table S2: baseline characteristics of the study population, Table S3: physical activity data, adherence to the Mediterranean diet, dietary fat intake and n-6/n-3 PUFA ratio in the four intervention groups, Table S4: effects of the intervention on arm circumference and tricipital skinfold in overweight/obese postmenopausal women.
