# Supplementary material for: Effects of DHA-Rich n-3 Fatty Acid Supplementation and/or Resistance Training on Body Composition and Cardiometabolic Biomarkers in Overweight and Obese Post-Menopausal Women
Source: Nutrients. 2021 Jul 19;13(7):2465. doi: 10.3390/nu13072465 (PMC8308734; doi:10.3390/nu13072465)
Supplement: Supplementary file 1 [file nutrients-13-02465-s001.zip › nutrients-1279955-supplementary.pdf]

nutrients

Supplementary Materials

Effects of DHA-Rich n-3 Fatty Acid Supplementation and/or Resistance Training on Body Composition and Cardiometabolic Biomarkers in Overweight and Obese Post-Menopausal Women

Elisa Félix-Soriano [1,2], Alejandro Martínez-Gayo [1,2], María José Cobo [1,2], Adriana Pérez-Chávez [1,2], Javier Ibáñez-Santos [3], Natalia Palacios Samper [3], Iñaki Goikoetxea Galarza [3], Marta Cuervo [1,2,4], Marisol García-Unciti [1], Pedro González-Muniesa [1,2,4,5], Silvia Lorente-Cebrián [1,2,4] and María J. Moreno-Aliaga [1,2,4,5,*]

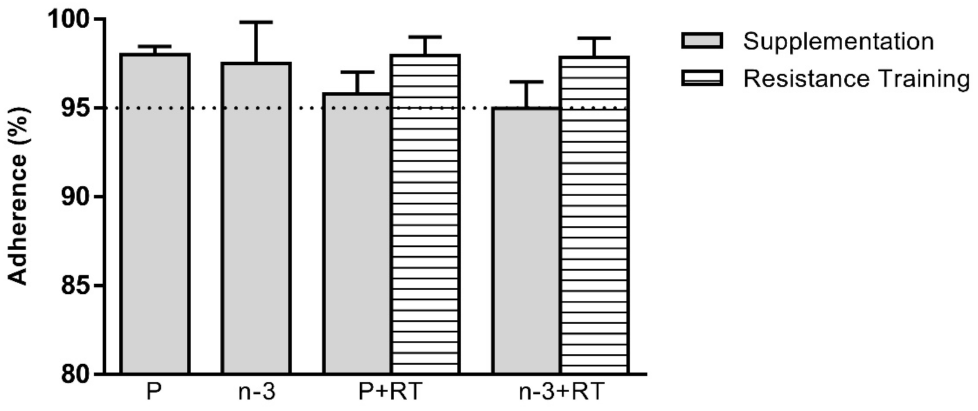

Figure S1. Adherence to supplements intake and resistance training program in the four intervention groups. P: placebo group, n-3: DHA-rich n-3 PUFA supplemented group; P+RT: placebo + resistance training group; n-3+RT: DHA-rich n-3 PUFA supplemented + resistance training group. Data are mean ± SEM.

Table S1. Fatty acid composition of the olive oil capsules used as placebo.

Fatty acid	(g/100 g fat)
Caprilic C8:0	0.09
Capric C10:0	ND
Lauric C12:0	ND
Myristic C14:0	ND
Palmitic C16:0	10.43
t-Palmitoleic C16:1	ND
Palmitoleic C16:1n-7	0.63
Stearic C18:0	2.78
Σ*Trans* isomers C18:1	0.32
Oleic C18:1 n-9	67.26
c-Vaccenic C18:1 n-7	1.85
t-Linoleic C18:2	ND
c-t Linoleic C18:2	0.24
t-c Linoleic C18:2	0.06
Linoleic C18:2 n-6	9.97
Arachidic C20:0	0.37
γ-Linolenic C18:3 n-6	ND
Eicosenoic C20:1 n-9	0.23
α-Linolenic C18:3 n-3	0.67
Stearidonic C18:4 n-3	ND
Eicosadienoic C20:2 n-6	0.11
Behenic C22:0	0.14
t-Brassidic C20:1	ND
Erucic C22:1 n-9	ND
Eicosatrienoic C20:3 n-3	ND
Arachidonic C20:4 n-6	ND
Eicopentaenoic C20:5 n-3	ND
Nervonic C24:1 n-9	ND
Docosatrienoic C22:3 n-9	ND
Docosapentaenoic C22:5 n-6	ND
Lignoceric C24:0	ND
Docosapentaenoic C22:5 n-3	ND
Docosahexaenoic C22:6 n-3	ND

Table S2. Baseline characteristics of the study population.

	P	n-3	P+RT	n-3+RT	
Number of subjects	20	15	20	16	*p* [a]
Age (years)	58.75 ± 3.39	58.00 ± 2.78	58.95 ± 3.46	58.13 ± 3.14	ns
Weight (kg)	76.75 ± 6.04	80.34 ± 8.51	77.76 ± 7.92	80.57 ± 6.60	ns
Height (m)	1.59 ± 0.06	1.62 ± 0.06	1.59 ± 0.06	1.61 ± 0.06	ns
BMI (kg/m^2)	30.25 ± 2.30	30.39 ± 1.94	30.79 ± 2.34	31.07 ± 1.82	ns
Fat mass (%)	47.44 ± 3.42	45.55 ± 2.38	47.05 ± 3.96	46.70 ± 2.90	ns
Lean mass (%)	49.76± 3.20	51.52 ± 2.18	50.16 ± 3.78	50.53 ± 2.76	ns
Visceral fat (kg)	1.30 ± 0.44	1.37 ± 0.44	1.27 ± 0.51	1.18 ± 0.47	ns
BMC (g)	2152.65 ± 308.05	2366.33 ± 332.76	2156.10 ± 231.21	2240.31 ± 258.36	ns
Waist circumference (cm)	93.11 ± 4.57	95.00 ± 7.63	92.67 ± 5.47	93.90 ± 7.16	ns
Hip circumference (cm)	110.68 ± 7.14	112.50 ± 5.78	110.65 ± 5.74	113.35 ± 6.82	ns
Waist/Hip Ratio	0.84 ± 0.04	0.85 ± 0.06	0.84 ± 0.06	0.83 ± 0.08	ns
Arms weight (kg)	8.65 ± 0.89	8.60 ± 1.17	8.73 ± 1.21	8.83 ± 1.04	ns
Arms fat mass (kg)	4.36 ± 0.60	4.01 ± 0.68	4.32 ± 0.68	4.24 ± 0.71	ns
Arms lean mass (kg)	4.02 ± 0.56	4.30 ± 0.60	4.13 ± 0.66	4.29 ± 0.47	ns
Legs weight (kg)	25.10 ± 3.59	27.52 ± 3.28	26.55 ± 3.91	27.11 ± 3.95	ns
Legs fat mass (kg)	11.29 ± 2.58	12.16 ± 2.38	12.36 ± 2.88	12.18 ± 2.47	ns
Legs lean mass (kg)	13.03 ± 1.57	14.51 ± 1.66	13.40 ± 1.64	14.11 ± 1.88	ns
Arm circumference (cm)	34.87 ± 1.49	34.02 ± 2.30	34.59 ± 2.31	35.56 ± 2.39	ns
Tricipital skinfold (mm)	31.85 ± 5.15	31.04 ± 3.91	32.95 ± 3.88	33.28 ± 5.42	ns
Thigh circumference (cm)	56.42 ± 4.54	56.75 ± 3.48	59.71 ± 5.21	60.42 ± 6.42	ns
Calf circumference (cm)	38.05 ± 2.12	39.59 ± 2.58	40.15 ± 2.41	39.30 ± 2.55	ns
Thigh skinfold (mm)	39.92 ± 4.76	41.25 ± 5.49	42.67 ± 2.99	41.06 ± 5.37	ns
Calf skinfold (mm)	31.07 ± 5.28	32.02 ± 5.60	33.26 ± 4.88	34.76 ± 5.45	ns
Lower limbs muscle strength	2.24 ± 0.38	2.26 ± 0.46	2.19 ± 0.40	2.01 ± 0.28	ns
Upper limbs muscle strength	0.44 ± 0.08	0.44 ± 0.10	0.45 ± 0.08	0.44 ± 0.05	ns
Lower limbs muscle quality	11.64 ± 3.38	11.86 ± 2.24	11.98 ± 1.81	10.89 ± 1.26	ns
Upper limbs muscle quality	7.40 ± 2.07	7.94 ± 1.59	8.05 ± 1.14	7.62 ± 0.99	ns
SBP (mmHg)	121.83 ± 19.68	119.18 ± 7.98	122.73 ± 14.96	123.67 ± 9.01	ns
DBP (mmHg)	80.04 ± 12.38	79.71 ± 6.05	79.08 ± 7.88	81.62 ± 6.67	ns
TG (mg/dL)	92.64 ± 29.47	118.20 ± 55.31	110.90 ± 51.66	101.34 ± 33.26	ns
Total Chol (mg/dL)	237.40 ± 30.79	239.73 ± 46.41	254.50 ± 27.83	250.31 ± 45.89	ns
LDL-Chol (mg/dL)	153.31 ± 32.65	154.20 ± 36.89	168.40 ± 24.49	164.90 ± 44.04	ns
HDL-Chol (mg/dL)	65.74 ± 16.77	61.89 ± 16.38	63.92 ± 14.61	65.15 ± 11.03	ns
Glucose (mg/dL)	98.57± 13.03 [1,2]	103.90 ± 15.72	109.14 ± 18.90 [1]	108.35 ± 11.32 [2]	0.034
AUC (mg min/dL)	15901.53 ± 4295.75	17942.80 ± 4904.76	17563.05 ± 4007.59	18006.69 ± 3890.92	ns
iAUC (mg min/dL)	4227.82 ± 3147.54	5652.67 ± 3787.17	5207.21 ± 3264.54	5260.25 ± 2934.61	ns
Insulin	10.02 ± 4.41	9.90 ± 5.21	9.49 ± 5.00	10.54 ± 4.05	ns
HOMA-IR index	2.51 ± 1.43	2.48 ± 1.19	2.67 ± 1.86	2.82 ± 1.14	ns
TyG index	8.45 ± 0.45	8.64 ± 0.41	8.64 ± 0.47	8.59 ± 0.39	ns
PA (METs-h/week) [b]	32.78 ± 17.26	22.65 ± 16.99	22.69 ± 25.08	19.99 ± 13.47	ns
MedDiet adherence (p14)	7.95 ± 1.91	8.13 ± 1.77	8.11 ± 2.06	7.81 ± 1.38	ns
Fat intake (g/day) [c]	124.36 ± 50.13	127.53 ± 45.54	141.74 ± 95.16	134.60 ± 57.25	ns
n-6/n-3 PUFA ratio [c]	5.67 ± 2.03	6.67 ± 4.45	5.26 ± 1.47	5.61 ± 2.27	ns

P: Placebo group, n-3: DHA-rich n-3 PUFA supplemented group; P+RT: Placebo + resistance training group; n-3+RT: DHA-rich n-3 PUFA supplemented + resistance training group; BMC: Bone mineral content; SBP: systolic blood pressure; DBP: diastolic blood pressure; TG: Triglycerides; Total Chol: Total Cholesterol; LDL-Chol: LDL-Cholesterol; HDL-Chol: HDL-Cholesterol; TyG: Triglycerides to Glucose index; PA: Physical Activity; MET: Metabolic equivalent of the task; MedDiet adherence: Mediterranean Diet adherence. Data are mean ± SD.

[a] Data were analyzed by one-way ANOVA or Kruskal-Wallis after testing for normality and homoscedasticity of the samples. Tukey or Dunn's multiple testing correction were used accordingly. [b] Data obtained from the validated physical activity questionnaire [46]. [c] Data obtained from the validated food frequency questionnaire [45].

[1, 2] Means that share a common superscript number were significantly different; ns, nonsignificant ($p > 0.05$).

Table S3. Physical activity data, adherence to the Mediterranean diet, dietary fat intake and n-6/n-3 PUFA ratio in the four intervention groups.

	**P**	**n-3**	**P+RT**	**n-3+RT**	**Two-way ANOVA** [c]		
N	20	15	20	16	n-3	RT	n-3xRT
PA (METs-h/week) [d]							
Baseline	32.78 ± 17.26	22.65 ± 16.99	22.69 ± 25.08	19.99 ± 13.47			
Change	3.15 ± 26.25	0.50 ± 14.56	4.90 ± 18.59	3.98 ± 10.88	ns	ns	ns
SPA (%) [e]	62.98 ± 11.16	61.91 ± 11.76	57.96 ± 11.72	58.21 ± 11.11	ns	ns	ns
LPA (%) [e]	29.48 ± 6.97	32.67 ± 8.50	31.50 ± 5.79	33.69 ± 6.96	ns	ns	ns
MPA (%) [e]	7.18 ± 7.04	5.37 ± 6.98	10.32 ± 10.54	8.07 ± 7.70	ns	ns	ns
VPA (%) [e]	0.37 ± 0.73	0.06 ± 0.13	0.23 ± 0.81	0.03 ± 0.08	ns	ns	ns
MedDiet adherence							
Baseline	7.95 ± 1.91	8.13 ± 1.77	8.11 ± 2.06	7.81 ± 1.38			
Change	1.55 ± 2.42 [a,**]	0.80 ± 2.11	0.50 ± 2.18	1.06 ± 2.18 [a,†]	ns	ns	ns
Fat intake (g/day) [f]							
Baseline	124.36 ± 50.13	127.53 ± 45.54	141.74 ± 95.16	134.60 ± 57.25			
Changes	-27.69 ± 39.68 [b,**]	-25.30 ± 31.42 [a,**]	-44.93 ± 92.79 [b,*]	-21.94 ± 35 [b,*]	ns	ns	ns
n-6/n-3 PUFA ratio [f]							
Baseline	5.67 ± 2.03	6.67 ± 4.45	5.26 ± 1.47	5.61 ± 2.27			
Changes	-0.52 ± 2.14	-1.25 ± 3.36	-0.89 ± 1.53 [a,*]	-0.67 ± 2.22	ns	ns	ns

P: Placebo group, n-3: DHA-rich n-3 PUFA supplemented group; P+RT: Placebo + resistance training group; n-3+RT: DHA-rich n-3 PUFA supplemented + resistance training group; PA: Physical Activity; MET: Metabolic equivalent of the task; SPA: sedentary physical activity; LPA: light physical activity; MPA: moderate physical activity; VPA: vigorous physical activity; MedDiet adherence: Mediterranean Diet adherence. Data are mean ± SD.

[a] Paired Student's *t*-test. [b] Wilcoxon's signed-rank test. [c] Differences in changes (16 weeks – baseline) between groups were evaluated by two-way ANOVA. [d] Data obtained from a validated physical activity questionnaire [46]. [e] Data obtained from 1-week recorded accelerometry. [f] Data obtained from the validated food frequency questionnaire [45].

** $p < 0.01$, * $p < 0.05$, † p trend ($p = 0.07$) vs. baseline; ns, nonsignificant ($p > 0.05$).

Table S4. Effects of 16 weeks of DHA-rich n-3 PUFA (n-3) supplementation and/or resistance training (RT) on arms anthropometric measurements in overweight/obese postmenopausal women.

	**P**	**n-3**	**P+RT**	**n-3+RT**	**Two-way ANOVA** [b]		
N	20	15	20	16	n-3	RT	n-3xRT
Arm circumference (cm)							
Baseline	34.87 ± 1.49	34.02 ± 2.30	34.59 ± 2.31	35.56 ± 2.39			
Change	-1.18 ± 1.32 [a,***]	-1.09 ± 0.82 [a,***]	-0.98 ± 0.96 [a,***]	-1.35 ± 1.16 [a,***]	ns	ns	ns
Adjusted change [c]	-1.22(0.21) [a,***]	-1.05(0.14) [a,***]	-1.01(0.20) [a,***]	-1.30(0.20) [a,***]	ns	ns	ns
Tricipital skinfold (mm)							
Baseline	31.85 ± 5.15	31.04 ± 3.91	32.95 ± 3.88	33.28 ± 5.42			
Change	-2.99 ± 4.31 [a,**]	-2.94 ± 2.88 [a,**]	-4.18 ± 3.69 [a,***]	-4.05 ± 3.49 [a,**]	ns	ns	ns
Adjusted change [c]	-3.35(1.05) [a,**]	-2.80(0.70) [a,**]	-4.05(0.85) [a,***]	-3.89(0.73) [a,***]	ns	ns	ns

DHA: Docosahexaenoic Acid; PUFA: Polyunsaturated Fatty Acids; P: placebo group; n-3: DHA-rich n-3 PUFA supplemented group; P+RT: placebo + resistance training group; n-3+RT: DHA-rich n-3 PUFA supplemented + resistance training group. Data are as mean ± SD.

[a] Paired Student's *t*-test. [b] Differences in changes (16 weeks – baseline) between groups were evaluated by two-way ANOVA. [c] Means(SEM) adjusted by changes in body weight.

*** $p < 0.001$, ** $p < 0.01$ vs. baseline; ns, nonsignificant ($p > 0.05$).
